# Individual Differences in Caregiving: Application of a Stress Process Model

**DOI:** 10.1093/geroni/igaa057.2276

**Published:** 2020-12-16

**Authors:** William Haley, Joanne Elayoubi

**Affiliations:** University of South Florida, Tampa, Florida, United States

## Abstract

Stress process models propose individual differences in caregiver outcomes depending on background characteristics and primary caregiving stressors, and resilience factors including stress appraisals, and internal and external resources. This paper will examine individual differences in the effects of the transition to caregiving on indicators of well-being and biomarkers of inflammation. Completed analyses show that, contrary to previous findings from cross-sectional studies, changes in well-being after caregiving generally do not differ by caregiver race, gender, age, or relationship category (spouse, adult child, others). Additional analyses examine the relationship of primary caregiving stressors (e.g. ADL and behavioral problems), stress appraisals (e.g., perceived stressfulness of ADL and IADL problems, perceived benefits of caregiving), and personality with changes in well-being and inflammation after the transition to caregiving. The lack of differences on most biomarker measures suggests that caregivers show substantial resilience in the face of significant, chronic caregiving stress.

